# Bodily sensations, emotions, and personality traits in the aesthetic experience of everyday photographs

**DOI:** 10.1038/s41598-025-29609-8

**Published:** 2025-12-26

**Authors:** Shoya Washizu, Yoshia Abe, Tatsuya Daikoku, Yasuo Kuniyoshi

**Affiliations:** 1 Graduate School of Information Science and Technology, The University of Tokyo, 7-3-1 Hongo, Bunkyo-ku, Tokyo, 113-8656 Japan; 2 Next Generation Artificial Intelligence Research Center, The University of Tokyo, 7-3-1 Hongo, Bunkyo-ku, Tokyo, 113-8656 Japan

**Keywords:** Computational biology and bioinformatics, Psychology, Human behaviour

## Abstract

Aesthetic evaluation is shaped by a complex interplay of bodily sensations, emotions, and personality traits. However, how these factors are correlated with aesthetic evaluation is not yet fully understood, and most prior work has centered on art-appreciation contexts. Here, we focus on everyday photographs to examine these relationships. In this study, 511 participants observed everyday photographs and reported bodily sensations; selected emotion categories from predefined labels; and rated valence, arousal, and aesthetic evaluation. Participants also completed five personality questionnaires assessing sensitivity to beauty, empathy, openness, alexithymia, and interoceptive awareness. The central finding was a robust association between higher aesthetic evaluation and chest-centered bodily sensations. Also, aesthetic evaluation was positively correlated with high-valence emotion categories and with higher levels of empathy and sensitivity to beauty. The contribution of this study lies in its multifaceted investigation of aesthetic experience grounded in everyday photographs. While broadly consistent with hypotheses derived from conventional art appreciation contexts, the results reveal partially distinct patterns of bodily sensations and emotional responses. Extending this line of inquiry to other aesthetic domains, such as music and morality, could help clarify how beauty arises.

**Supplementary Information:**

The online version contains supplementary material available at 10.1038/s41598-025-29609-8.

## Introduction

Humans are capable of evaluating the degree of beauty in various objects, events, and concepts. Aesthetic experience is a phenomenon shared by most individuals, playing a significant role in shaping our cultural and social foundations. Through aesthetic experience, we arrive at an aesthetic evaluation of how beautiful something we encounter feels to us.

 We focus on “everyday photographs”—images primarily taken by amateurs that depict scenes and people rooted in daily life. As Leder et al. first pointed out an important element when conceptualizing aesthetic experience as a cognitive process, aesthetic experience is not necessarily limited to traditional forms of art such as historical paintings, music, film, or poetry^1^. Although these photographs were not necessarily produced or collected with the intention of eliciting aesthetic experience, examining their beauty is important in at least two respects.

 The experience of viewing everyday photographs can be understood as an aesthetic experience accompanied by everydayness, distinct from the appreciation of artworks such as paintings, and one that may yield unique insights into aesthetics. Two considerations motivate this focus. First, the everyday quality of photographs may evoke an aesthetic sense. In the field of philosophical aesthetics, “everyday aesthetics” explores the relationship between daily life and beauty. It is argued that beauty can be discovered within ordinary scenes and phenomena, such as the sense of comfort evoked by a familiar street corner or the functional beauty of tools grounded in daily life^2,3^. Second, photography can be seen as a cultural activity that has its own artistic qualities. According to van Dijck, even when photographs are not intended for aesthetic appreciation, the choices involved in framing, composition, and subject matter often reflect implicit aesthetic evaluation^4^. The emergence of particular aesthetic styles within photographic culture—exemplified by what is sometimes referred to as “Instagram aesthetics”^5^—resembles stylistic changes in painting, but with the noteworthy difference that such trends extend beyond professional photographers to include amateur image-makers^6^. This highlights the need to attend not only to aesthetic evaluation as it pertains to viewers, but also to the aesthetic evaluation exercised by individuals as photographers engaging in daily acts of image-making. Taken together, viewing photographs of everyday scenes constitutes a distinct form of aesthetic experience, separate from the appreciation of traditional artworks. Accordingly, employing such image stimuli to study aesthetic experience is expected to yield new insights into the relationships among beauty, emotion, and bodily responses.

 This study comprises analyses from three perspectives: bodily sensations, individual traits, and emotions. Viewing everyday photographs can be framed as an aesthetic experience in which bodily sensations play a central role: aesthetic experience comprises emotional and bodily processes, including valence as an affective dimension and aesthetic chill, respiratory changes, and heart-rate fluctuations as bodily sensations. In this everyday context, such bodily sensations may be especially pronounced. The VIMAP model—extending Leder et al.’s cognitive-process account—explicitly incorporates bodily sensations^7^. Moreover, VIMAP characterizes aesthetic chill (a bodily sensation associated with aesthetic experience) as correlating with personal relevance (e.g., domain knowledge or prior art exposure) and emerging when expectations of harmony or emotion are fulfilled. Thus, images with high personal relevance—such as familiar landscapes or animals—are likely to elicit similar bodily sensations. In parallel, body map studies of art/media-art appreciation have reported head-centered bodily sensations associated with aesthetic evaluation^8^. Inspired by these two lines of work, we focus on bodily responses. On this basis, we hypothesized that harmony rooted in everyday photographs would distinctively shape beauty and bodily sensations, and we examined their distribution using the body map, a quantitative method for assessing subjective bodily sensations, to enable comparison with prior work in other modalities.

Next, we consider the relationship between emotions and aesthetic experience. While aesthetic experience is expected to relate to emotions with high valence, it need not be confined to positive emotions; it may also relate to negative emotions such as sadness. By examining correlations among a broad set of emotions (beyond aesthetic experience), aesthetic evaluation, valence, and arousal, we aim to characterize in detail the emotions that co-occur with aesthetic experience when viewing everyday photographs.

 Finally, we conducted questionnaire-based investigations of individual differences from two viewpoints: first, we examined the relationship between aesthetic experience and bodily sensations in everyday photographs. Prior work suggests that aesthetic chill correlates with higher openness to experience^9^. Accordingly, we hypothesized that greater openness would correlate with higher subjective ratings of aesthetic experience and with greater reported bodily sensations. We also compared our results with multiple prior body map studies to assess whether the distribution of bodily sensations varies with individual traits. Second, we conducted tests grounded in prior theories and models as well as our own hypotheses. Freedberg et al. argue that viewing artworks can evoke embodied, emotion- and action-based empathy^10^. In contrast, Kesner and Horáček (2017) contend that a mirror-neuron–based account is insufficient^11^; instead, they emphasize cognitive efforts to understand the depicted figures or creators, proposing that empathic responses to artworks arise from interactions among social, emotional, and cognitive processing and the processes of aesthetic appreciation and judgment. We hypothesized that individuals with higher empathy would report higher aesthetic evaluation and greater bodily sensations. We also expected that higher sensitivity to beauty would be associated with increased reports of bodily sensations, and we therefore measured sensitivity to aesthetic experience.

In summary, to evaluate aesthetic experience related to everyday photographs, this study comprised three analyses: body map (analysis 1), emotions (analysis 2), and personality traits (analysis 3). These analyses were conducted within a single session using the same participants and the same dataset.

 In analysis 1, to analyze bodily sensations experienced when viewing images, we employed the body map. The body map is a technique for quantitatively analyzing subjective bodily sensations and has recently gained attention for linking a range of concepts—such as emotions, morality, and love—with subjective physical experiences^12–14^. Daikoku et al. reviewed existing body map studies and proposed a conceptual framework consisting of three components: physiological signals, behavioral engagement, and conceptual and metaphorical constructions^15^. More recently, some studies have applied the body map to visual aesthetic experience. Schino et al., for instance, demonstrated that the evaluation of artworks is correlated with increased activity in the head region^8^.

Using the body map, we investigated how the distribution of bodily sensations varies with differences in aesthetic evaluation of everyday photographs. Specifically, we examined the correlation between each participant’s aesthetic evaluation and both the overall intensity and the relative intensity across body regions reported in the body map. We then compared how these intensities varied with the perceived beauty of the images, referencing prior findings based on other types of stimuli.

In analysis 2, we evaluated emotional responses to everyday photographs. Aesthetic evaluation can be understood as a form of emotion, and by focusing on emotional components, we aim to better situate aesthetic evaluation within the broader spectrum of emotional responses.

 In this study, using the valence–arousal model and emotion categories, we investigated correlations between individual emotional responses and aesthetic evaluation. Based on the theoretical framework in which valence is defined as the degree of pleasantness–unpleasantness when viewing an image and arousal as the intensity or activation level of the emotional response^16,17^, we measured valence, arousal, and aesthetic evaluation (a beauty scale) for each photograph as integrated indices using a 9-point Likert scale. For emotion categories, we adopted 32 categories following the taxonomy proposed by Keltner and Lerner^18^.

In analysis 3, to gain a more detailed understanding of how personality traits relate to aesthetic evaluation and body map indices, we examined correlations among five personality questionnaires and the valence and arousal ratings.

 First, to explore the relationship between bodily activity reported via the body map and aesthetic experience involving physiological responses and bodily empathy—such as aesthetic chill—we employed three self-report questionnaires. We administered the Engagement with Beauty Scale (EBS), which measures sensitivity to aesthetic experience in domains such as nature, art, and morality; the short version of the Multidimensional Empathy Scale (MES)^19,20^; and the Japanese version of the Ten Item Personality Inventory (TIPI-J)^21^. Using these measures, we explored the relationship between bodily sensations reported on the body map and aesthetic experience, including physiological responses (e.g., aesthetic chill) and embodied empathy. To evaluate how individual differences in empathy for different kinds of aesthetic experience influence body map responses, we used a Japanese translation of the EBS (Engagement with Beauty Scale). We hypothesized that individuals with higher EBS scores—indicating greater sensitivity to aesthetic experience—would show higher aesthetic evaluation. Additionally, Freedberg et al. have argued that viewing artworks can evoke embodied emotional and behavioral empathy^10^. To investigate the connection between the body map and bodily simulation based on empathic traits, we employed a short version of the Multidimensional Empathy Scale (MES)^19,20^. We hypothesized that individuals with higher MES scores—reflecting stronger empathic tendencies—would exhibit more intense bodily sensations on the body map. Furthermore, among physiological responses, aesthetic chill—often triggered during the appreciation of artworks—is known to be positively correlated with the personality trait of openness^22^. In the present study, we used the Japanese version of the Ten Item Personality Inventory (TIPI-J)^21^ to assess the Big Five personality traits and examined their correlations with body map data, with particular attention to openness.

 Second, using the Toronto Alexithymia Scale (TAS-20) to assess alexithymic tendencies and the Body Perception Questionnaire–Body Awareness subscale (BPQ-BA VSF-J) to assess sensitivity to interoceptive signals, we compared prior body map findings with data from the present study. Multiple studies employing auditory stimuli have shown between-group differences in body map activity. Daikoku et al. demonstrated that individuals with higher alexithymic tendencies exhibit a more diffuse body map distribution^23^. Building on this evidence, we hypothesized that individuals scoring high on certain personality traits would report more bodily sensations; we then examined whether BPQ-BA VSF-J^24^ and TAS-20^25^ scores correlated with reported bodily sensations. Tanaka et al. further reported that individuals with high BPQ-BA VSF-J scores exhibit significantly stronger bodily sensations in the chest and abdominal regions than those with low scores^26^.

We conducted two complementary analyses. First, we performed multi-modal prediction using ResNet-50 with measured emotion labels and body map labels (Fig. S1, S2, S3, S4). Second, we performed composite aesthetic evaluation prediction using LLM-generated pseudo-labels (Fig. S5). Details of the methods and results are provided in the Supplementary Materials.

This study focuses on aesthetic experience in everyday contexts that are not readily captured by prior research centered on artworks. Across our analyses, we integrated analyses of bodily responses, emotional evaluations, and individual traits to advance understanding in this domain. Specifically, drawing on body map data, emotion ratings, and personality measures, we clarified how emotions relate to bodily responses in moments of strong aesthetic experience and identified the factors that correlate with individual differences in aesthetic experience.

## Results

Analysis 1: Evaluation of the Relationship Between Subjective Bodily Sensations and Aesthetic Evaluation Using the Body Map.

To investigate the relationship between bodily sensations and beauty, we conducted two types of analyses: a distribution analysis of bodily sensations based on participants’ subjective evaluations and a correlation analysis using the average values for each image. All results are available on OSF (https://osf.io/e9wx2/).

### Intensity plot on the body map

**Fig. 1:**
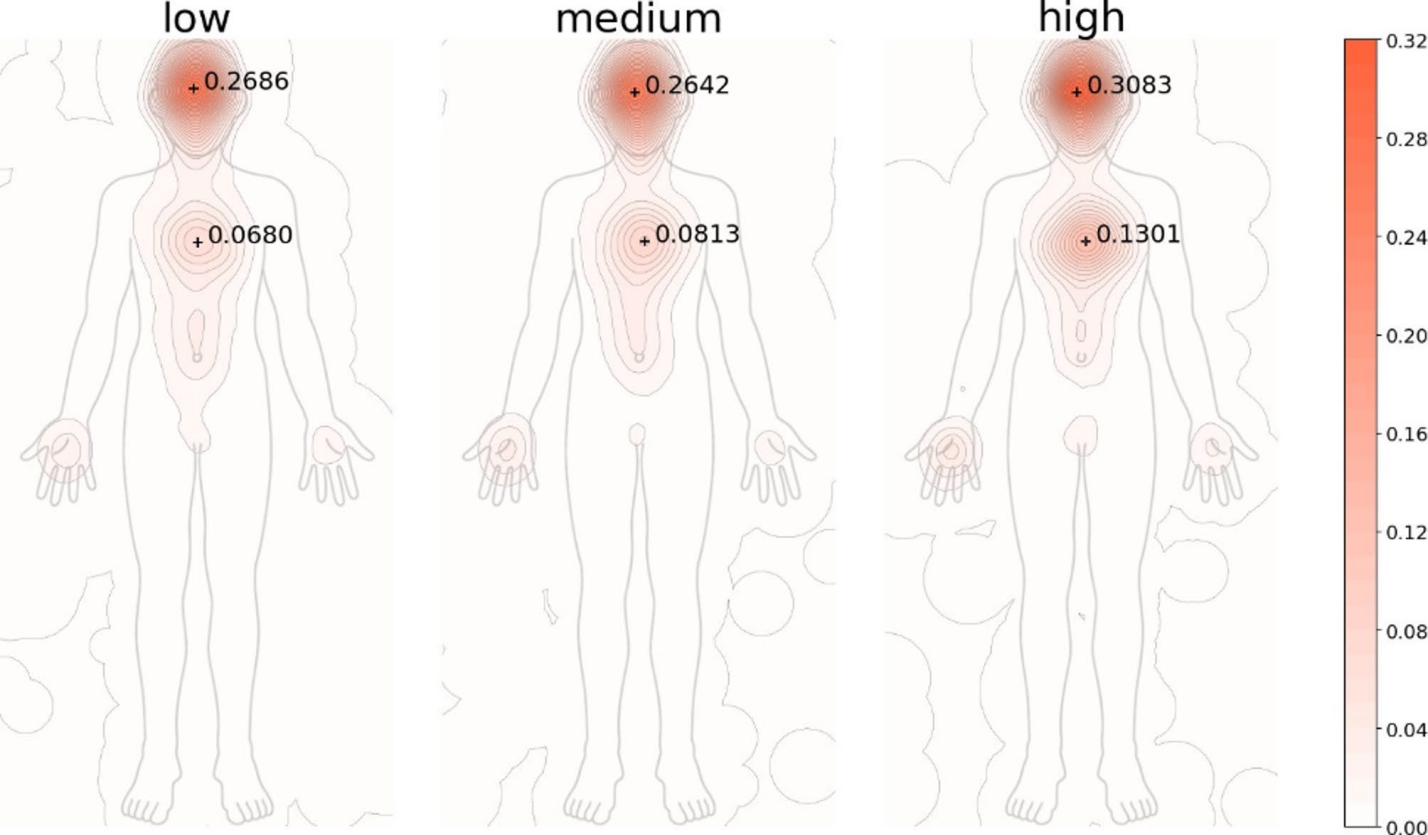
Relationship between beauty evaluation and bodily sensations. The nine-point beauty rating scale was divided into three groups, and for each group, the relative intensity of bodily sensations obtained through body map was visualized. The labels in the figure indicate the peak intensity positions and relative values for clusters observed in the head and chest regions.

Figure 1 displays the intensity of bodily sensations, categorized into three rating levels—High (7 ∼ 9), Medium (4 ∼ 6), and Low (1 ∼ 3). The color intensity in the plot indicates the relative strength per trial, with darker regions indicating stronger bodily sensations in that body region.

Overall, the results reveal stronger bodily sensations in the head and chest regions. Examining the peak intensity in these areas shows that bodily sensations in both the head and chest are particularly pronounced in the High-rating group. This suggests that participants who gave higher aesthetic evaluation tended to report more bodily sensations in these regions on the body map.

### Results of correlation analysis

**Fig. 2:**
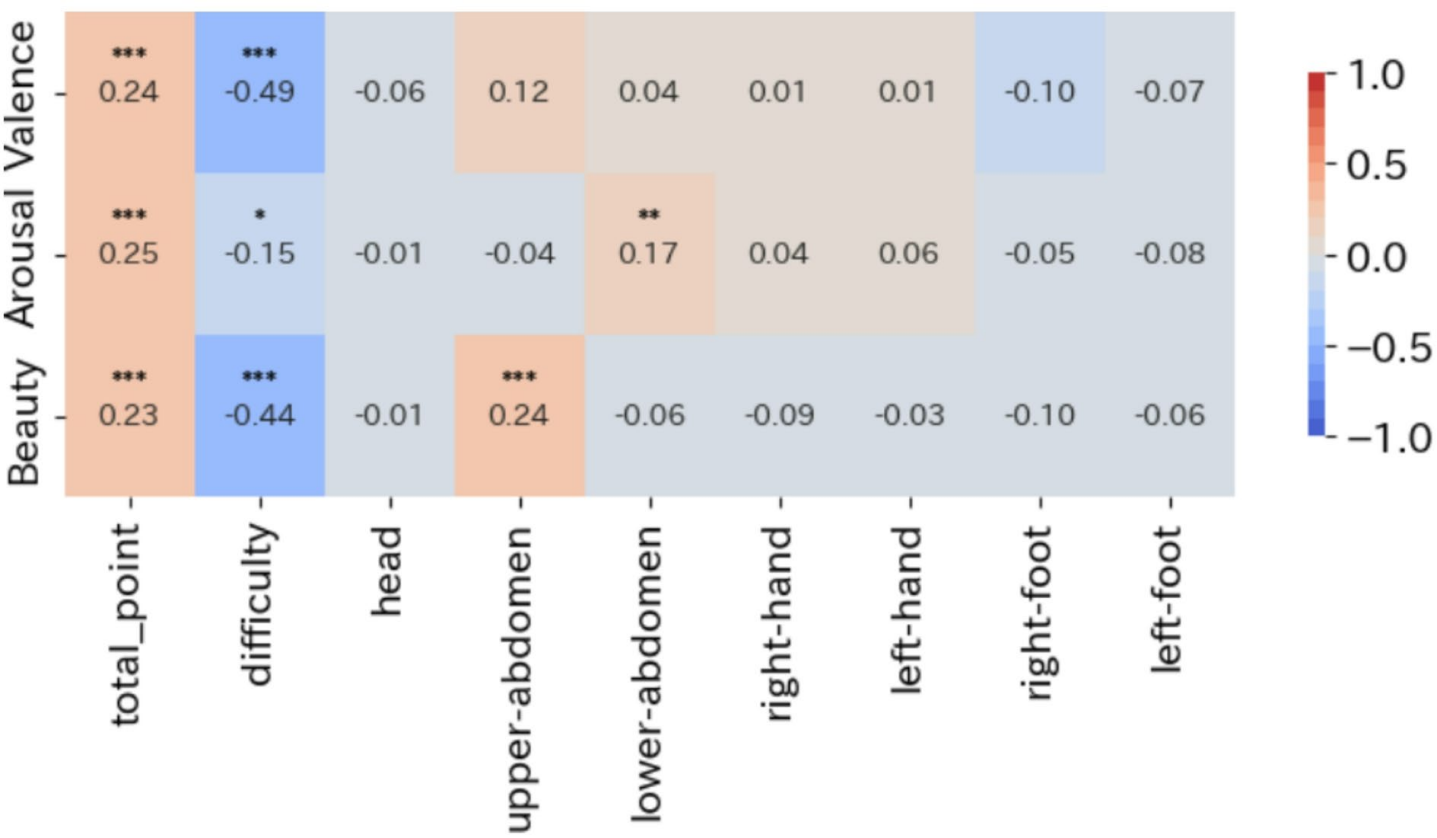
Relationship between body map, valence, arousal, and beauty evaluation. Correlation analysis was performed using Spearman’s rank correlation coefficient, with valence, arousal, and beauty evaluation plotted on the vertical axis and body map evaluations by body region, averaged per image, plotted on the horizontal axis. Correlation tests were conducted for each item in the table to assess the significance of correlations among these indicators. To account for multiple comparisons, FDR correction was applied. Each correlation coefficient is marked with asterisks corresponding to the FDR-corrected q-values, as follows: (*q* < 0.001: ***, *q* < 0.01: **, *q* < 0.05: *). A strong correlation (*q* < 0.001) was observed between the difficulty of selection and the proportion of bodily sensations in the chest region, in relation to aesthetic evaluation.

Figure 2 presents the correlation relationships between the average evaluation of valence, arousal, and beauty for each image and the body map metrics—proportion by body region, intensity of bodily sensations, and average rating of selection difficulty—for each image.

Significant correlations (*q* < 0.001) were observed between aesthetic evaluation and sensations in the chest region, as well as between arousal and sensations in the abdominal region. Additionally, a trend was observed across all items indicating that higher evaluation were associated with an increased intensity of bodily sensations. This aligns with the overall stronger bodily sensations seen across body map intensity plots. While the intensity was generally higher in both the head and chest regions in the plotted data, the correlation analysis based on body-region proportions of bodily sensations indicated that images with higher aesthetic evaluation were particularly associated with a greater proportion of bodily sensations mapped to the chest region.

### Analysis 2: evaluation of the relationship between emotion labels and aesthetic evaluation

This section presents the results of correlation analysis to examine the relationships among emotion labels, aesthetic evaluation, and each level of emotional valence.

**Fig. 3:**
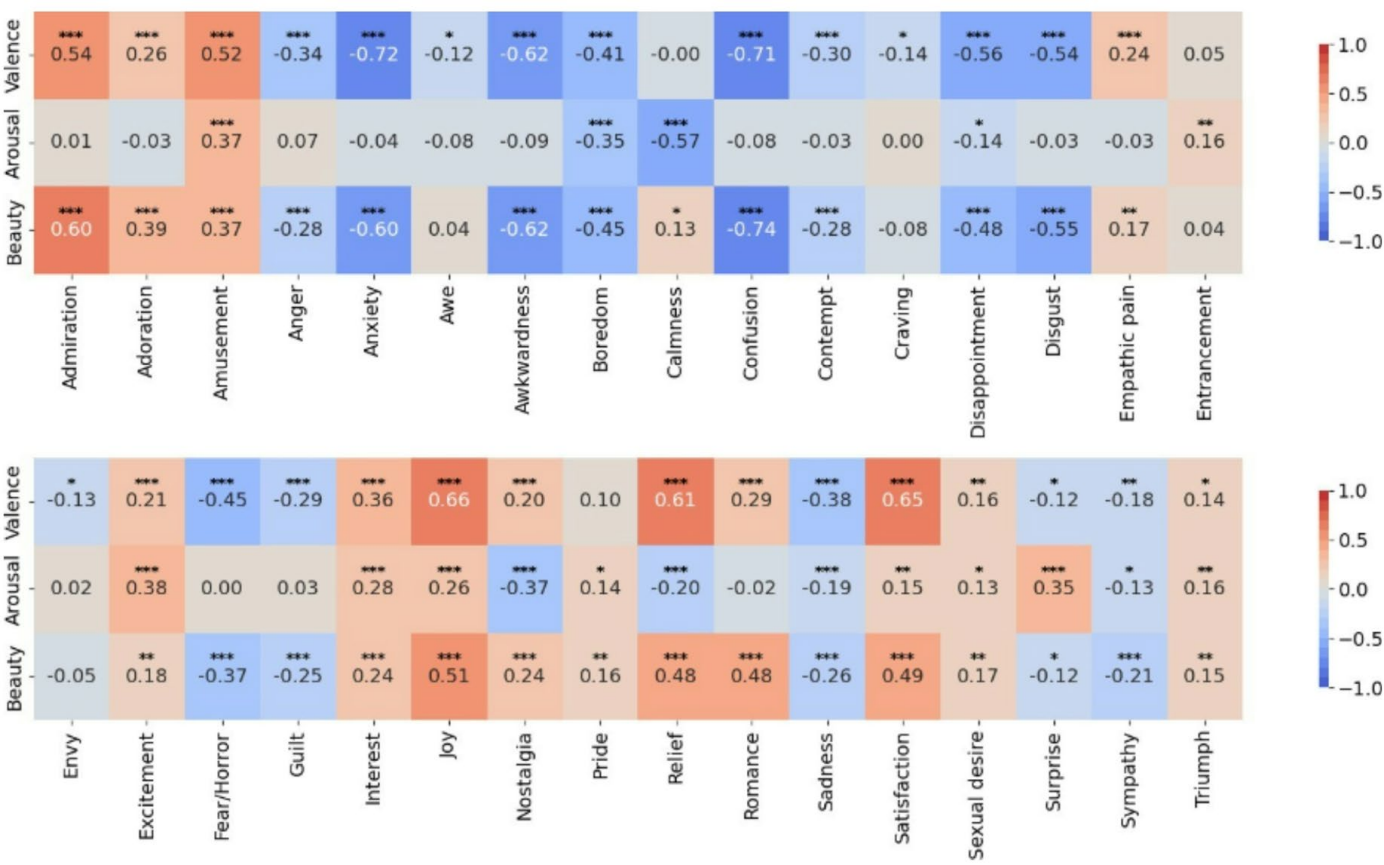
Relationships among 32 emotional labels and image evaluation of valence, arousal, and beauty. The vertical axis represents the evaluation scores for valence, arousal, and aesthetic quality, while the horizontal axis represents the evaluation scores for each of the 32 emotional labels used in the analysis. The data was averaged for each image, and Spearman’s rank correlation coefficient was employed to investigate correlations among the variables. Additionally, we conducted correlation tests for each item in the table to assess the significance of correlations among the indicators. False Discovery Rate (FDR) correction was applied to adjust for multiple comparisons. Correlation coefficients are annotated with significance markers based on the FDR-corrected q-values as follows: (*q* < 0.001: ***, *q* < 0.01: **, *q* < 0.05: *). Results indicated a significant relationship (*q* < 0.05) between aesthetic quality and 28 of the 32 emotional labels.

Figure 3 illustrates the extent to which each emotion label affects emotional valence, arousal, and beauty for each image.

All results are available on OSF (https://osf.io/e9wx2/).

Most emotions were found to be significantly correlated (*q* < 0.05) with valence, arousal, and beauty. Furthermore, there was an observed trend indicating that valence and beauty share similar correlation patterns.

### Analysis 3: relationships among body map, aesthetic sensitivity, and participant characteristics

To examine the relationships among personality traits, body map characteristics, and evaluations of beauty, we conducted a correlation analysis to investigate how the intensity of bodily sensations and the participants’ average beauty evaluation were influenced by specific personality traits. All results are available on OSF (https://osf.io/e9wx2/).

**Fig. 4:**
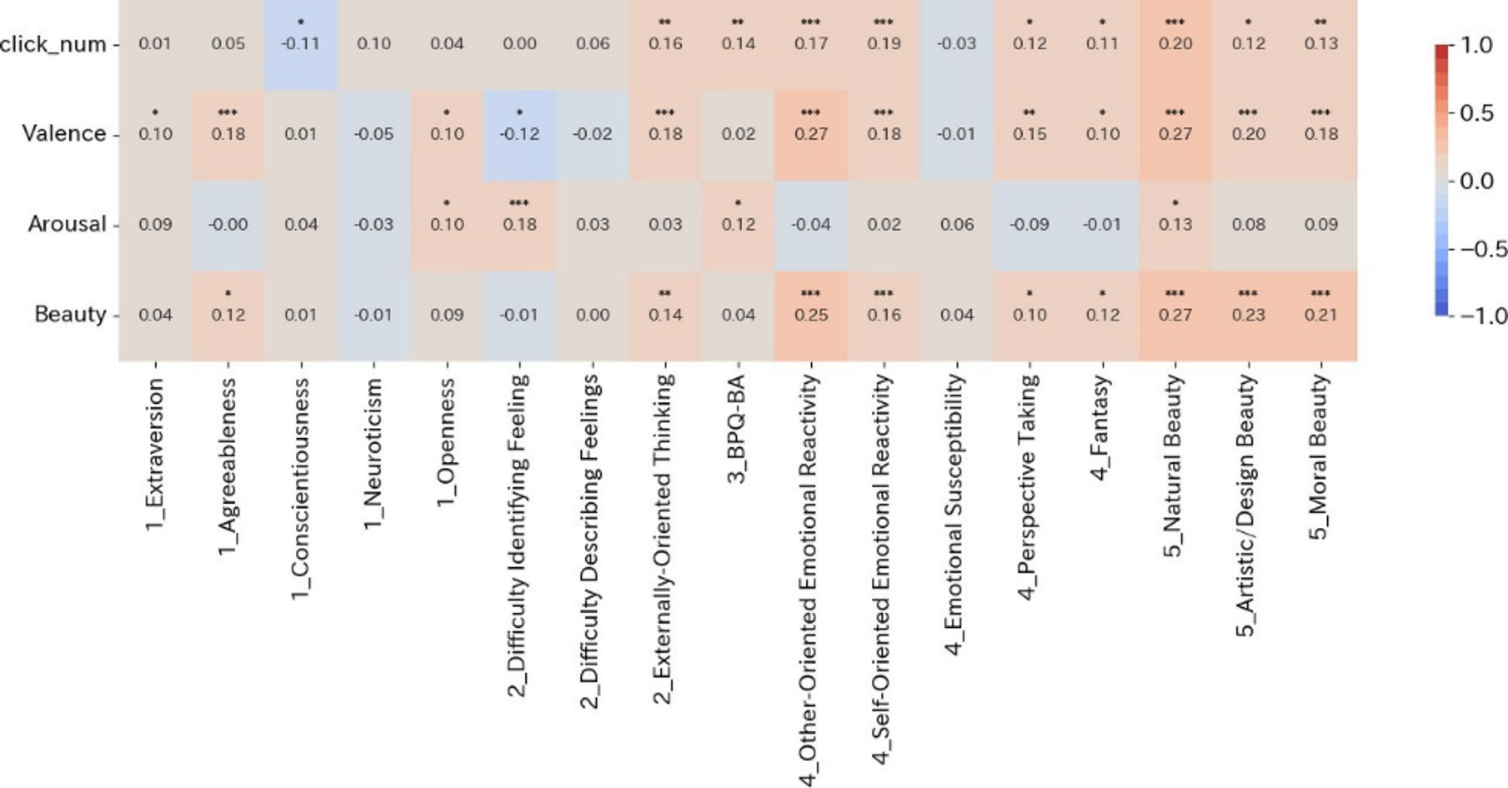
Relationship between bodily sensations, emotions, aesthetics, and personality traits by participant. The vertical axis represents the mean scores for bodily sensation, valence, arousal, and beauty evaluation for each participant, and the horizontal axis represents the scores for the individual questionnaires. Spearman’s rank correlation coefficient was used to analyze these relationships. Correlation tests were conducted for each item in the table to evaluate the significance of correlations between indicators. FDR correction was applied for multiple comparisons, and correlation coefficients were annotated with asterisks based on FDR-adjusted q-values (*q* < 0.001: ***, *q* < 0.01: **, *q* < 0.05: *). Strong correlations (*q* < 0.001) were observed between aesthetic sensitivity and both the MES (a measure of empathy) and the EBS (a measure of aesthetic sensitivity), particularly in relation to aesthetic evaluation.

Figure 4 presents the results of this correlation analysis. Focusing on the intensity of bodily sensations and beauty, positive correlations were observed with all items of the EBS, which assesses sensitivity to beauty, with four of the five MES items (which measure empathy, excluding susceptibility to influence), and with the externally oriented thinking item of the TAS-20, a questionnaire for assessing alexithymia. In contrast, different trends were observed with the TIPI-J, which assesses the Big Five personality traits, and with the BPQ-BA VSF-J, which measures sensory interoceptive awareness. Within the TIPI-J, correlations were found between agreeableness and beauty, and negative correlations were found between honesty and the intensity of bodily sensations. While no significant correlation was found between interoceptive awareness and beauty, a moderately strong correlation (0.01 ≦ *q* < 0.05) was observed between interoceptive awareness and the intensity of bodily sensations.

### Summary and relation to prior studies

#### These findings support or diverge from several hypotheses derived from prior research

- Body map: While previous studies hypothesized an correlation between aesthetic experience and bodily activity in the head region, the present results revealed that increased aesthetic evaluation was accompanied by greater activity in the chest region.
- Body map and bodily sensitivity (BPQ-BA VSF-J): As hypothesized, bodily sensitivity was positively associated with the intensity of bodily sensations, reflecting a consistent relationship between BPQ-BA VSF-J scores and overall body map intensity.
- Body map and alexithymia (TAS-20): Although previous studies indicated more diffuse body map distributions among individuals with high alexithymia, the present study observed a correlation with the intensity of bodily sensations, thus only partially aligning with earlier findings.
- Aesthetic chill (TIPI-J): While openness showed a correlation with valence, it was not associated with aesthetic evaluation or body map activity. In contrast, stronger correlations were found with other traits such as agreeableness, contradicting the initial hypothesis.
- Empathy (MES) and bodily sensations associated with aesthetic experience (EBS): Consistent with prior expectations, both aesthetic evaluation and the intensity of bodily sensations were positively associated with measures of empathy and subjective bodily awareness, supporting the hypothesis that bodily empathy and subjective evaluation play a role in aesthetic experience.

In addition to the results presented here, we conducted three additional analyses, reported in the Supplementary Materials: a UMAP visualization of the emotion point cloud (Fig. S6); a frequency analysis of body-map click counts (Fig. S7); and correlations between physical condition and aesthetic evaluation (Fig. S8). Simple code for the body-map visualization is provided as Algorithm 1 in the Supplementary Materials.

## Discussion

This study shows that (i) body map responses to everyday photographs are concentrated in the head and chest and increase in magnitude with higher aesthetic evaluation, (ii) beauty aligns closely with positive valence while a subset of discrete emotions diverges from this trend, and (iii) trait measures related to aesthetic sensitivity and empathy predict higher beauty ratings and stronger bodily reporting, whereas alexithymia and interoceptive awareness differentially track arousal and amount of bodily sensation. Together, these findings indicate that everyday images—not only canonical artworks—reliably elicit embodied and affective components of aesthetic experience.

 In analysis 1, the head–chest emphasis and its scaling with beauty are consistent with prior body map research on love^13^. Using auditory stimuli, Daikoku et al. further linked chest activity to positive emotional states^27^. Extending these observations, we found that higher aesthetic evaluation was associated with both a global increase in bodily intensity and a relative concentration in the chest region. However, this pattern differed from the head-centered activity reported for visual/media art^8^. In the present experiment, bodily sensations in the head region were pronounced regardless of the image’s level of aesthetic evaluation, and no correlation was observed between aesthetic evaluation and the intensity of head-region bodily sensations. This pattern suggests that, in the aesthetic evaluation of everyday photographs, intuitive processing exerts a greater influence than logical reasoning such as reflection or cognition about artworks. To further discuss the extent of intuitive/emotional processing, additional experimental verification is required. Increases in bodily sensations in the head and chest also point to links with aesthetic chill and with the verbal recognition of bodily reactions. The upper abdomen includes the lungs and heart; phenomena such as gasping at something beautiful or feeling one’s heartbeat quicken are plausibly reflected as bodily sensations^28^. In the present participant study, responses on the body map were obtained before participants reported aesthetic evaluation and emotion labels for each image. Although this ordering does not entirely eliminate meta-cognition through verbalization, it is noteworthy that common verbal expressions describing reactions to beauty were quantitatively recapitulated as body map responses. Furthermore, sensations extending from the chest to the abdomen intensified as aesthetic evaluation increased, hinting at a potential link with “aesthetic chill,” which is often perceived as sensations along the back.

The insights of the present study are confined to responses to everyday photographs, which constitute a limitation. Although the present study focused specifically on everyday photographs, future research comparing body map responses across multiple types of artworks—such as historical paintings, abstract art, and media art—may help clarify whether everyday photographs evoke bodily sensations that are distinctively different. Such comparisons could contribute to a deeper understanding of which types of artworks are more closely correlated with bodily sensations associated with aesthetic experience.

 In analysis 2, for each image, participants selected their top three perceived emotions, and we examined correlations between highly rated images and aesthetic evaluation. According to the correlation analysis (Fig. 3), emotions that tracked valence also tracked aesthetic evaluation—positively and negatively alike. These results indicate that, in general, higher valence—that is, pleasantness—is closely related to perceived beauty. This pattern is consistent with prior work on aesthetic experience in response to paintings^29^, which reported a positive correlation between positive emotions and beauty. Some emotions, however, displayed different tendencies for valence and aesthetic evaluation. Awe, Craving, and Envy showed significant negative correlations with valence, yet no significant correlation with aesthetic evaluation. One possible interpretation is that Craving and Envy involve the absence or unattainability of a desirable object, which reduces pleasantness even when the object’s value or appeal is judged high, whereas Awe may evoke fear and respect that can coexist. These hypotheses require further experimental testing. Significant correlations between arousal scores and emotion scores followed intuitively plausible patterns: arousal tended to be higher for Amusement, Excitement, Interest, Joy, and Surprise, and lower for Boredom, Calmness, Nostalgia, Relief, and Sadness. Notably, even when participants rated arousal and discrete emotions separately for a single image, we obtained consistent tendencies.

 Several emotions diverged from findings in prior research on aesthetic experience, potentially due to properties of the stimuli or the relative rarity of certain phenomena. Awe is often discussed in relation to aesthetic experience^7^, yet our analysis did not reveal a significant positive correlation with aesthetic evaluation. Regarding Sadness, although the notion of “the beauty of sorrow”^30^ suggests that sadness and beauty can co-occur, the present study observed a significant negative correlation with aesthetic evaluation. These outcomes may reflect the fact that our stimuli consisted of photographs of everyday scenes rather than artworks and that the co-occurrence of aesthetic experience with awe or sorrow may not be common.

 In analysis 3, trait analyses (Fig. 4) show correlations between the subjective strength of perceived beauty and empathy: all three EBS subscales (nature, art/design, moral beauty) correlated positively with beauty, and multiple MES-SF facets (other-/self-oriented emotional response, perspective taking, fantasy) showed similar relationships, consistent with the idea that emotional/imaginative attunement enhances aesthetic evaluation^10^. In other words, scores on a questionnaire assessing sensitivity to beauty were positively associated with the aesthetic evaluation scores measured in our experiment, a result that supports the validity of the present findings. Among the personality subscales of the MES-SF (Multidimensional Empathy Scale, short form), scores for other-oriented emotional response, self-oriented emotional response, perspective taking, and fantasy each showed positive correlations with aesthetic evaluation. These results are consistent with the interpretation that emotional and imaginative attunement to the target of appreciation can enhance aesthetic evaluation, and they support the argument that empathic engagement underlies art appreciation^10^.

 By contrast, other personality traits did not show consistent correlations. Of the Big Five traits measured by TIPI-J, Agreeableness showed a significant positive correlation with aesthetic evaluation—again, a trait related to empathy. Based on prior work indicating that individuals high in Openness are more prone to aesthetic chill^22^, we expected a significant positive correlation with Openness; however, this was not observed. Possible explanations include that “intensity of beauty”/“aesthetic pleasantness” and “occurrence of chills” may reflect different phenomena, that our stimuli consisted of everyday photographs rather than artworks, and the limited precision of the short-form scale (TIPI-J). Alexithymic tendencies were characterized more by their relation to arousal than to aesthetic evaluation. On TAS-20, externally oriented thinking correlated positively with aesthetic evaluation, whereas “difficulty identifying feelings” and “difficulty describing feelings” showed no significant correlation with aesthetic evaluation. In contrast, “difficulty identifying feelings” showed a strongly significant positive correlation with arousal. That is, individuals who have difficulty identifying their emotions tended to report higher arousal. Although the causal direction remains to be examined, one possible mechanism is that identifying one’s emotions may help attenuate arousal. Awareness of interoceptive sensations was related to the strength of self-reported bodily responses but not to aesthetic evaluation per se. Scores on BPQ-BA VSF-J, which assesses the degree of awareness of interoceptive sensations, did not correlate significantly with aesthetic evaluation but did show a positive correlation with the number of clicks reported on the body map. In other words, individuals with higher interoceptive awareness tended to report more bodily responses—a reasonable and expected pattern.

Through this experiment, we clarified relationships among people’s aesthetic evaluations of photographic images and their bodily sensations, emotions, and personality traits. Regarding sensations, we found a positive correlation in the upper abdominal region where the lungs and heart are located; as emotions, we generally observed positive correlations with emotion categories of higher valence; and regarding personality traits, we found positive correlations with higher empathy. Although the mechanisms generating these correlations cannot be specified within the scope of the present results, future research that systematically integrates diverse forms of aesthetic experience—including artworks, music, and morality—may help elucidate these relationships.

## Methods

This study conducted an analysis using data obtained from a participant-based experiment. Participants were recruited via an online experimental service. Before the experiment, participants reported having no history of mental illness or visual impairment. Subsequently, participants were asked to complete a body map task using test images prepared in advance to ensure the experiment could be conducted as intended. The experiment was conducted in accordance with the guidelines of the Declaration of Helsinki and was approved by the Ethics Committee of The University of Tokyo (Screening number: UT-IST-RE-230710). Additionally, informed consent was obtained from all participants at the beginning of the experiment, and no questions were included that could reveal personally identifiable information. A total of 511 participants, both male and female, aged between their 20s and 60s, took part in the experiment. Among them, 238 were female, and 2 participants did not disclose their gender. The majority of participants (N = 509) were in their 20 s to 40 s.

The experiment is broadly divided into two main parts: a section investigating individual characteristics using questionnaires, and a stimulus evaluation section using images.

### Investigation of individual characteristics using questionnaires

Based on several previous studies, we selected questionnaires likely to be relevant to the subjective representation of bodily sensations depicted through images. These questionnaires were designed to investigate correlations between participants’ body map responses and variations in aesthetic sensitivity. The questionnaires used in the experiment are presented in Table 1. All participants completed the five questionnaires in the order listed in Table 1, and the order of items within each questionnaire was not altered.

**Table 1:** Description of the questionnaire used in the experiment.

Questionnaire	Content
TIPI-J^21^	10-item scale of the big five personality traits
TAS-20^25^	3-factor measure of alexithymia
BPQ-BA VSF-J^24^	Scale of internal sensory hypersensitivity
MES^20^	5-dimension empathy scale
EBS^31^	Scale of three domains of beauty sensitivity

 The Big Five personality traits, include five distinct factors, and several questionnaires have been developed to analyze these traits^32^. A strong correlation has been observed between bodily experiences involving aesthetic sensitivity and openness, one of the Big Five factors^22^. This study examined whether similar characteristics could be reproduced when using the body map. For this experiment, a Japanese-adapted short form of the Big Five Inventory (BIG5), consisting of 10 items, was used^21,33^. It should be noted that prior studies have conducted evaluations using the more detailed BFAS questionnaire, which comprises 100 items and assesses 10 sub-factors^34^.

 This study also examines the relationship to previous research using the body map. Daikoku et al. demonstrated that, when listening to sounds, individuals with alexithymia or depression exhibit a more diffuse distribution in their body map responses^23^. Furthermore, studies using the BPQ-BA, a subscale of the Body Perception Questionnaire (BPQ)^35^ designed to assess subjective bodily experiences, have also suggested a potential correlation with the body map^26^. Against these backgrounds, this experiment employed a Japanese translation of the TAS-20, a questionnaire for assessing alexithymia^25,36^, and a Japanese-translated short form of the BPQ-BA (BPQ-BA VSF-J)^24,37^. This approach aimed to investigate whether modulations in bodily sensations similarly occur in aesthetic evaluations of images.

 Furthermore, although prior research suggesting that the brain’s empathetic responses to bodily states are correlated with aesthetic perception^10^, the relationship between the body map and empathy has been relatively unexplored. This study aimed to investigate the connections between empathy in response to stimuli, aesthetic perception, and the body map. For this purpose, we conducted a survey using a shortened version of the Multidimensional Empathy Scale (MES)^19,20^.

 Finally, as a questionnaire related to aesthetic perception, we employed the EBS^31^, which measures the intensity of aesthetic experiences related to nature, art, and morality, after translating it into Japanese for use in this study.

For the four questionnaires other than the EBS, we used Japanese translations of the survey items that had been previously published in academic papers. Prior to the image-stimulus task, participants completed these five questionnaires.

### Image evaluation

 In the image evaluation section, participants were presented with 18 images selected from a set of 347 photographs in the PARA dataset^38^. The PARA dataset includes basic participant characteristics (such as Big Five personality traits), along with ratings of image attributes like aesthetic appeal and brightness, as well as scene labels for each image. The 18 images used in this study were chosen to represent six primary scenes in the dataset, with an equal distribution of three aesthetic rating levels based on average aesthetic scores for each image.

 For each image, participants first completed the body map assessment together with a difficulty rating for this task. They then evaluated emotional valence, arousal, and beauty using a 9-point Likert scale, and finally ranked the top three emotions evoked by the image from a set of 32 emotional labels. The emotional labels used for the affective evaluation were adapted from the 33 emotion labels employed in Cowen et al.’s experiment^39^, with all labels except “beauty” translated into Japanese for this study. For the body map assessment, participants were asked to click on areas of the body where they sensed a reaction while viewing each image, with multiple clicks on the same area indicating stronger sensations. Additionally, participants rated the difficulty of this task using a 9-point Likert scale.

Analysis 1: Evaluating the Relationship Between Subjective Bodily Sensations and Beauty Using Body Map To investigate the relationship between subjective bodily sensations and perceptions of beauty, we conducted an analysis focusing on the proportion of each body part in body map plots.

To determine the intensity of sensation for each body part per image, we counted the number of points plotted within each region and calculated the proportion relative to the total number of clicks across all trials. For the correlation analysis, we used these proportions, along with the average values of each image’s valence evaluations and selection difficulty, to calculate correlation coefficients.

The measurement of significant differences for each of the analyses, including analyses 2 and 3, is corrected by multiple testing. Spearman’s rank correlation coefficient was employed to investigate correlations among the variables. Additionally, we conducted correlation tests for each item in the table to examine the significance of the relationships among the indicators. False Discovery Rate (FDR) correction was applied to adjust for multiple comparisons. Correlation coefficients are annotated with significance markers based on the FDR-corrected q-values as follows: (*q* < 0.001: ***, *q* < 0.01: **, *q* < 0.05: *).

We also conducted an analysis that visualizes the points plotted on the body map. Participants’ evaluations were categorized into three levels—High (7 ∼ 9), Middle (4 ∼ 6), and Low (1 ∼ 3)—with shading applied to indicate the locations on the body map that correspond to each level. The size (intensity) of the circles overlaid on the body map reflects the number of points.

each participant plotted. To ensure consistency, the radius of each circle was adjusted so that participants with a higher number of plotted points and those with fewer had circles of equal intensity and area. Pseudo-code for the program used in this analysis is provided in the supplementary algorithm S1.

### Analysis 2: evaluation of the relationship between emotional labels and aesthetic perception

To examine the relationship between emotions evoked by the images and aesthetic ratings, we conducted correlation analyses. This analysis incorporated the top three emotional labels rated by participants for each image, as well as the 9-point Likert scale ratings for valence, arousal, and aesthetic perception. For each image, we calculated the average emotional labels based on the ranked emotional labels provided by participants, representing the emotions evoked by each image as a vector across the dimensions corresponding to the number of emotional labels.

In the correlation analysis, we calculated correlation coefficients using the weighted emotional label values derived for each image as described above, along with the average valence ratings for each image. In the analysis, it is important to note that the correlation being examined is not between the label values for each individual trial and the evaluations within those trials. Rather, average values for each image are used in place of trial-specific data.

### Analysis 3: evaluating the relationship between body map, aesthetic perception, and personality traits

In the preparatory phase of the experiment, several questionnaires were selected based on prior research to assess individual characteristics (refer to “Investigation of Individual Characteristics Using Questionnaires”). Using these questionnaires, we conducted a correlation analysis to examine how participants’ personality traits influenced their image evaluations and body map responses. For each participant, we computed the average values of valence, arousal, beauty ratings, and the number of clicks on the body map across 18 evaluated images. We then calculated the correlations between these averages and the scores of each personality trait.

## Supplementary Information

Below is the link to the electronic supplementary material.

Supplementary Material 1

## Data Availability

Data on the results of subject experiments can be provided upon reasonable request. The photographic data presented to the subjects is based on the PARA dataset^38^ and should be referred to the authors of the paper.

## Code availability

The code for the analysis in this study can be provided upon reasonable request.
