# Supplementary material for: Bodily sensations, emotions, and personality traits in the aesthetic experience of everyday photographs
Source: Sci Rep. 2025 Dec 26;16:348. doi: 10.1038/s41598-025-29609-8 (PMC12770382; doi:10.1038/s41598-025-29609-8)
Supplement: Supplementary file 1 — Supplementary Material 1 [file 41598_2025_29609_MOESM1_ESM.docx]

# Supplementary information

# Supplementary Experiment: Multidomain Prediction of Aesthetic Judgment Using LLMs

# Exp. A-1: Applicability to Aesthetic Judgment Prediction Tasks

In this study, we collected not only aesthetic judgment scores for individual images but also associated emotional labels and Body Map data. In this section, we examine whether these additional modalities are useful for predicting image aesthetic judgment, a task commonly referred to as Image Aesthetics Assessment [1][2]. Specifically, we focus on the Generic Image Aesthetics Assessment (GIAA), which involves predicting the average aesthetic judgment scores across multiple participants for a given image.


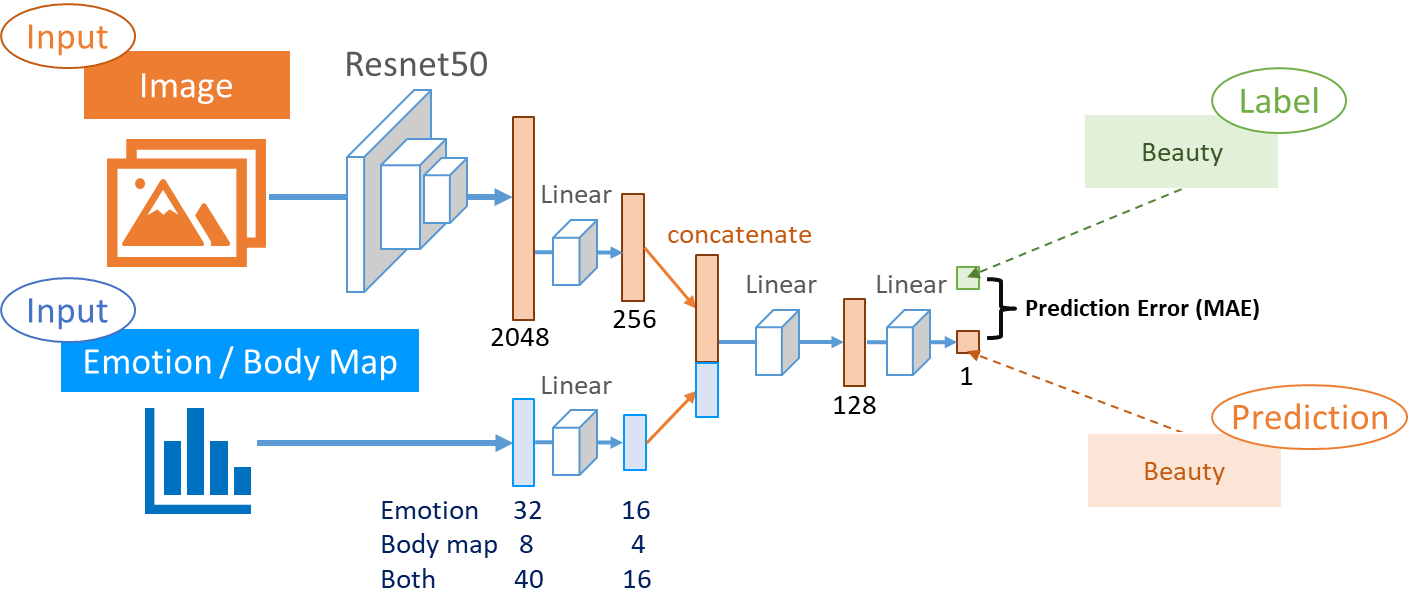


**Figure S1** Overview of the model used in experiments to predict the aesthetic evaluation of an image using Resnet50.

We constructed a deep neural network model as illustrated in Fig. S4.
 The image input branch uses ResNet-50 [3] to extract visual features from the image, followed by a linear layer.
 The emotional labels and Body Map data are each passed through separate linear layers to generate feature vectors. These vectors are then concatenated with the image features to form a combined representation, which is used to predict the final aesthetic judgment score.

During training, the network was optimized using the mean absolute error (MAE) between the predicted and ground-truth scores. The ResNet-50 weights were initialized using pretrained parameters.

**Encoding Emotional Label Information**


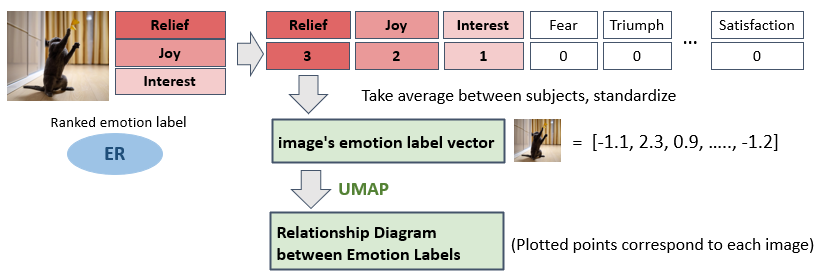


**Figure S2** Schematic illustration of preprocessing for UMAP-based structural analysis. An image from the PARA dataset used in the actual experiment [4] is shown as part of the figure. erview of the model used in experiments to predict the aesthetic evaluation of an image using Resnet50.

In each trial, participants were asked to select the top three emotions they felt in response to a single image, from a set of 32 predefined emotional labels. A weighted scoring system was applied, assigning 3, 2, and 1 points to the top 1, top 2, and top 3 selected emotions, respectively. This produced a 32-dimensional vector for each participant per image. Since each image was rated by multiple participants, we computed the average across all participants’ vectors to obtain a single 32-dimensional vector representing the overall emotional distribution for that image. This vector was used as the input for emotional labels.

**Encoding Body Map Information**


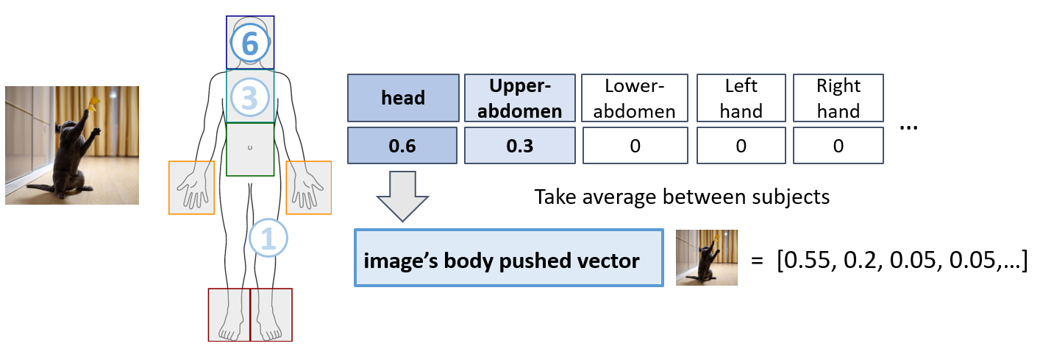


**Figure S3** Schematic illustration of the calculation method for regional proportions in the Body Map. An image from the PARA dataset used in the actual experiment [4] is shown as part of the figure.

In each trial, participants reported the regions of the body where they felt sensations in response to a given image. As illustrated in Fig. S6, the body was divided into seven predefined regions. For each trial, we calculated the proportion of clicks in each of the seven regions and included the total number of clicks as an eighth value. This resulted in an 8-dimensional vector per participant per image. The final input vector for the Body Map was obtained by averaging these vectors across participants for each image.


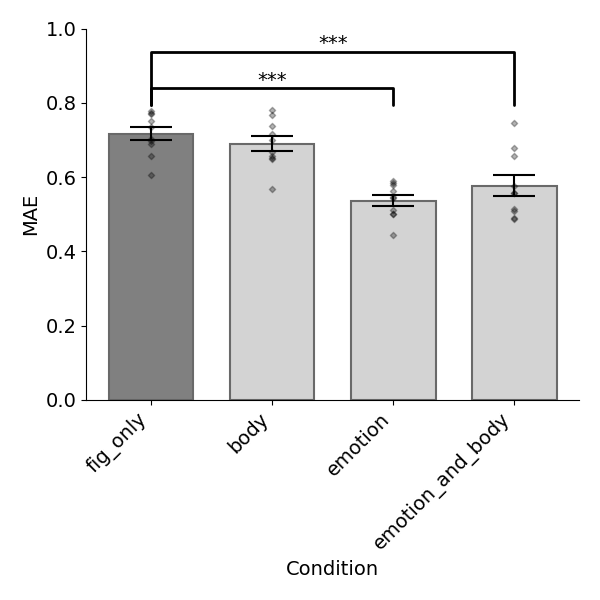


**Figure S4** Domains Used for Training and Prediction Accuracy. This analysis evaluated the mean prediction error and standard deviation for aesthetic ratings across ten prediction tasks using 50 test image data. Predictions were made under four conditions: using image data alone, image data with Body Map information, image data with emotion label information, and image data with both Body Map and emotion label information. Dunnett's test ($\alpha<0.001$) was used for significance testing, with the image-only condition serving as the control group; conditions with significant differences from the control are marked with three asterisks.

**The experimental procedure and results**

Among the 347 images presented to participants, 50 were randomly selected as test data, and the remaining 297 were used for training. To account for variation, the process of splitting the training and test sets was repeated using 10 different random seeds.

The experiment compared the following four input conditions:

- fig_only: Only the image was used as input.
- body: The image and Body Map information were used as input.
- emotion: The image and emotional label information were used as input.
- emotion_and_body: The image, emotional label information, and Body Map information were all used as input.

The results are shown in Fig. S7.
As shown in the figure, conditions using emotional labels (emotion and emotion_and_body) achieved significantly higher prediction accuracy compared to the image-only baseline.

These results suggest that emotional label information contributes meaningfully to the prediction of aesthetic judgment. Notably, even with a lightweight input method in which participants selected only their top three emotions, the resulting emotional vectors proved useful for aesthetic judgment prediction. This finding is encouraging, especially given the high cost typically associated with building large-scale aesthetic judgment datasets.

On the other hand, Body Map information did not significantly improve prediction accuracy. One possible explanation is that the number of clicks on the Body Map varies substantially between individuals, making the averaged data less informative for the Generic Image Aesthetics Assessment (GIAA) task, which focuses on predicting mean ratings. It is possible, however, that Body Map data may be more useful in the context of Personalized Image Aesthetics Assessment (PIAA), where individual-level predictions are required. Exploring this possibility is left for future work.

# Exp. A-2: Applicability to Aesthetic Judgment Prediction Tasks (LLM-Based Substitution)

In the previous section, we used participants' reported emotional labels and Body Map information as additional inputs to the prediction model alongside the image. However, in practical applications where predicting aesthetic judgment is necessary, it is often difficult to obtain actual emotional or bodily responses to an image.

Therefore, instead of using participant-provided emotional labels or Body Map data, it is desirable to utilize pseudo-emotional and pseudo-bodily information predicted directly from the image using some automated method.

To this end, we employed Large Language Models (LLMs) to simulate human-like responses, using them to predict emotional labels and Body Map information from the image alone. Specifically, we used GPT-4o, which accepts image inputs, as our LLM. In place of multiple human participants, we conducted three separate query trials per image and used the aggregated responses. The temperature was set to 0 to ensure deterministic outputs.

# **Method for Obtaining Emotional Labels**

# We adopted two methods to acquire emotional label information from the LLM:

- **Emotion1**: The LLM was prompted to select the top three emotions from a predefined set of 32 emotional labels in response to a given image. Scores of 3, 2, and 1 were assigned to the top-1, top-2, and top-3 emotions, respectively. The resulting 32-dimensional vectors from three independent trials were averaged to produce a final emotional label vector for each image.
- **Emotion2**: The LLM was prompted to rate the intensity of each of the 32 emotions on a 9-point Likert scale for the given image. Again, responses from three trials were averaged to produce a final emotional label vector for each image.

### **Method for Obtaining Body Map Information**

The LLM was prompted to assign a non-negative integer value representing the intensity of bodily sensation for each body region in response to a given image. Responses from three independent trials were averaged to produce an 8-dimensional Body Map vector for each image.

**The experimental procedure and results**

Due to a failure to obtain responses from the LLM in all three trials for one image, we used the remaining 346 images in this experiment. Using the pseudo-emotional labels and Body Map vectors generated by the LLM, we trained the same prediction model as in Exp. A-1 (Fig. S4). As before, 50 images were randomly selected as test data, and the remaining 296 were used for training. To account for variation, we repeated the train/test split and training process using 10 different random seeds.

### **Input Conditions for Comparison**

The following four input conditions were compared in this experiment:

- **Figure**: Only the image was used as input.
- **Fig_Emotion1**: The image and pseudo-emotional labels obtained via Emotion1 were used as input.
- **Fig_Emotion2**: The image and pseudo-emotional labels obtained via Emotion2 were used as input.
- **Fig_Body**: The image and pseudo-Body Map information were used as input.

The results are shown in Fig. S8.
 As illustrated in the figure, the model trained using pseudo-emotional labels from the Emotion2 method achieved significantly higher prediction accuracy compared to the image-only baseline.

These results reinforce the importance of emotional information in predicting aesthetic judgment. Furthermore, they suggest that GPT-4o possesses a sufficiently high capacity for emotion prediction, to the extent that it may serve as a viable proxy for human emotion evaluation. This raises the possibility of bypassing the need for human-generated emotion data in aesthetic judgment prediction tasks.
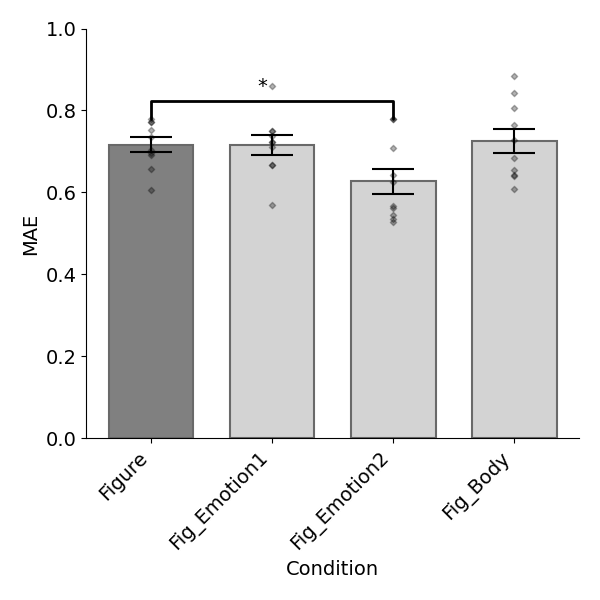


**Figure S5** Results of Aesthetic Judgment Prediction Using LLM-Generated Pseudo-Emotion and Pseudo-Body Map Labels. Predictions were made under four conditions. Dunnett's test ($\alpha<0.05$) was used for significance testing, with the image-only condition serving as the control group; conditions with significant differences from the control are marked with an asterisk.

**Example of prompts used in the experiment**

Q1:

Can you imagine specific body parts corresponding to the sensations triggered by this image?

The list of body parts is: head, upper abdomen, lower abdomen, right hand, left hand, right foot, left foot.

For the part where you feel a sensation, answer with an integer larger than 0.

If the sensation is strong, provide a larger number.

...

Q2:

Please rate the difficulty of the previous task on a scale from 1 (easy) to 9 (difficult).

...

Q3:

Please rate your impression of the presented image on a 9-point scale.

Valence (1 = unpleasant/negative, 9 = pleasant/positive)

Arousal (1 = calm/relaxed, 9 = excited)

Beauty (1 = ugly/not beautiful at all, 9 = very beautiful)

...

Q4:

Please rank the emotions you feel from the presented image by selecting top three different items.

You cannot select the same item for different ranks.

Here are the emotion options: Admiration, Adoration, Amusement, Anger, Anxiety, Awe, Awkwardness, Boredom, Calmness, Confusion, Contempt, Craving, Disappointment, Disgust, Empathic pain, Entrancement, Envy, Excitement, Fear/Horror, Guilt, Interest, Joy, Nostalgia, Pride, Relief, Romance, Sadness, Satisfaction, Sexual desire, Surprise, Sympathy, Triumph

....

# Visualization of the Gradient of Point Clouds on UMAP Using Least Squares Plane Regression

The vector values for the emotional labels were determined as follows. During the image evaluation phase, participants selected the top three emotional labels they felt most strongly represented each image. A weighted intensity score was assigned to each label: a score of 3 for the top-ranked label, 2 for the second, and 1 for the third. These scores were then compiled into a vector for each trial, and the average vector values were calculated across participants who evaluated the same image.

To capture the relationships between the tendencies of emotion labels assigned to each image and their evaluations of valence, arousal, and beauty, we conducted an analysis using dimensionality reduction. Specifically, we applied UMAP as the dimensionality reduction technique, projecting the emotion labels for each image onto a two-dimensional plane. On this two-dimensional representation, we visualized the average evaluations for valence, arousal, and beauty for each image using color intensity, with the darkness indicating the relative magnitude of each evaluation.

In the UMAP experiment, a least squares plane regression was applied to approximate the point clouds by a plane, where the (x, y) plane corresponds to the structural plane of the UMAP, and the z-axis represents the values of each point. This approach was used to quantitatively analyze the directional alignment of the Valence and Arousal axes. In Figure S1, the vectors depicted on each plot represent the projection of the normal vectors of the fitted planes onto the (x, y) plane. Using this method, we quantitatively identify the optimal direction of the vector under the assumption that Valence and Arousal vary monotonically along a specific axis on the UMAP structural plane.

# Frequency Histogram of Body Map Clicks

Figure S2 illustrates the frequency with which each participant clicked on the Body Map (the horizontal axis represents the total number of clicks across 18 images). The data reveal that a subset of participants concentrated their clicks within a limited range. Humans are capable of evaluating the degree of beauty in various objects, events, and concepts. Aesthetic sensibility is a quality shared by most individuals, playing a significant role in shaping our cultural and social foundations. However, the cognitive mechanisms that evoke aesthetic experiences remain largely unknown.

# Physical Condition and Emotions

In the section evaluating individual characteristics during the participant experiment, we also investigated aspects subject to variation over time or due to state changes, including a question about the participants' current physical condition. This was assessed using a 9-point Likert scale. The results are shown in Figure S3. Since this question was experimental and not part of the formal questionnaire, it has been analyzed separately from the main analysis.

# Algorithm Used for Body Map Visualization

To visualize the frequency of clicks on different regions of the Body Map, we represented the data as variations in color intensity over the body image used in the experiment. This approach is commonly employed in analyses of experiments utilizing similar tests. In this study, considering the need to account for individual differences in click frequency and to examine variations in the clicked regions across beauty evaluations, we employed a custom algorithm for the visualization. Thus, previous studies have suggested that aesthetic perception involves not only inherent emotions but also personal traits and bodily sensations. However, much remains unknown about the precise mechanisms underlying these interactions.

Algorithm S1 succinctly represents the function used for visualizing the Body Map. This algorithm was employed to generate the visualizations presented in Figure 3 of the main paper.


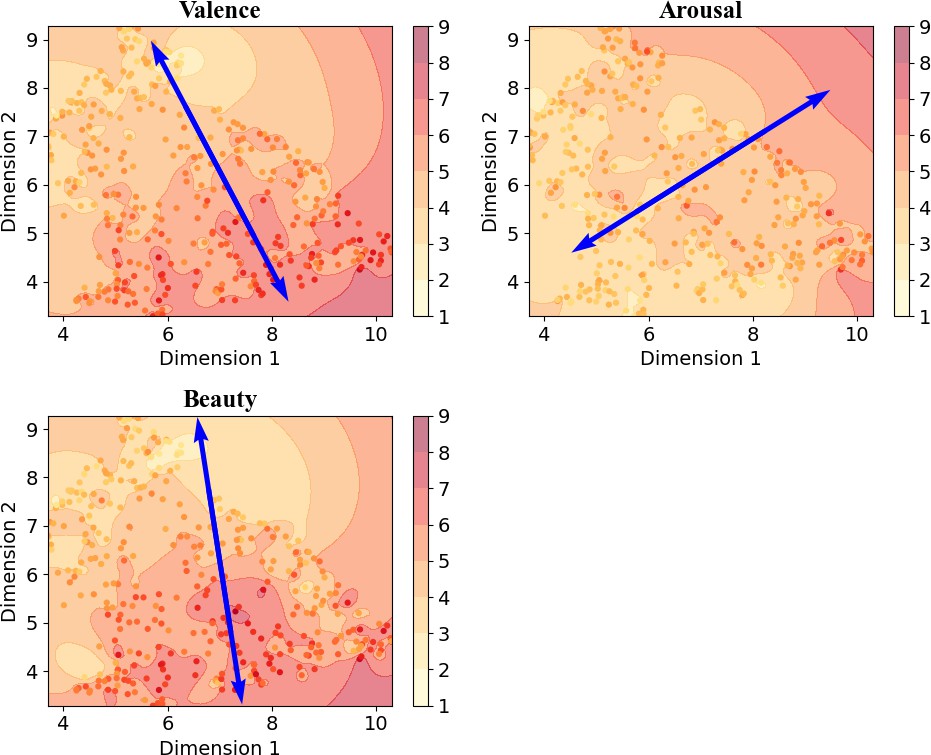


**Figure S6.** Approximation Axes for Each Metric in Structural Analysis of Emotion Labels Using UMAP


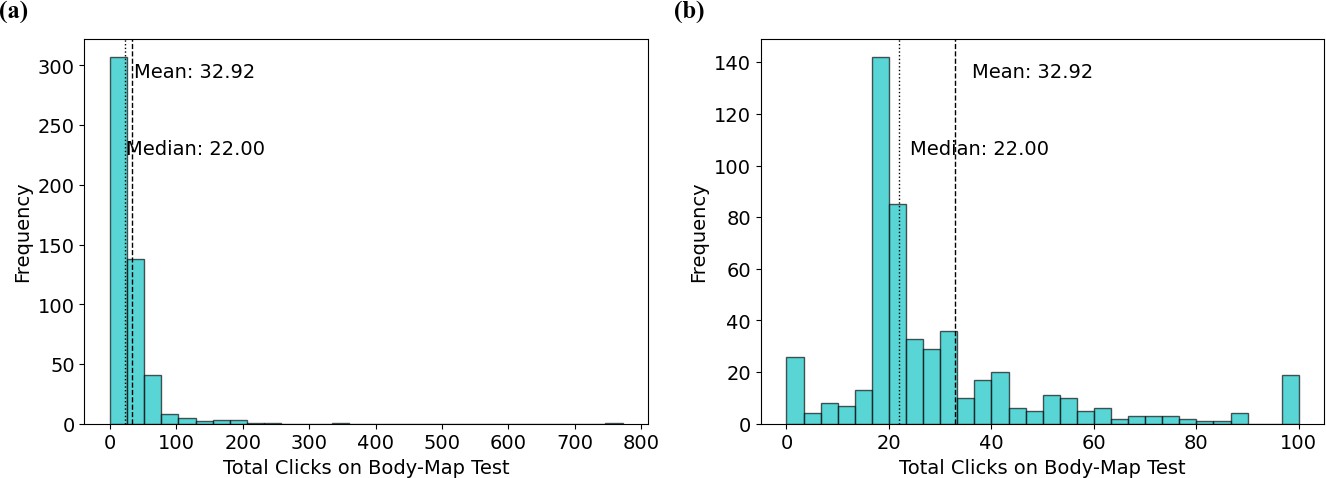


**Figure S7.** Distribution of Click Counts (a) Represents the distribution results for all participants. (b) Shows a cropped version of (a), limited to the range of 0 to 100.


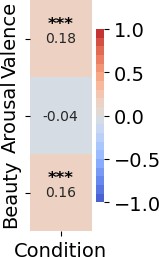


**Figure S8** Relationship Between Condition and Emotions The vertical axis represents the evaluation scores for Valence, Arousal, and Beauty, while the horizontal axis shows the results of the question about physical condition. Correlation analyses were conducted, and the p-values for each correlation coefficient are indicated as follows: *** for p<0.001, ** for p<0.01, and * for p<0.05.

**Algorithm S1** Body Map Visualization Function

**Require:** *ALL_POINT*: has all click data, each click contains:

*1: (p.X, p.Y )*: where clicked*,*

*2: p.CLICK*: Total number of click times of the subject on all image,

*3: p.VALUE*: which beauty value participant choose in this image

*4:* (1 ∼ 3=low, 4 ∼ 6=mid, 7 ∼ 9=high),

*5: (IMAGE_X_PIXEL, IMAGE_Y_PIXEL)*: Body-Map image size,

*6: (RADIUS, INTENSITY)*: graphing circle base size and intensity

***Ensure*:** img:graph of the Body Map test intensity

*7:* ***function*** Draw_Circle *(x, y, clicknum)*

*8: circle_center ← (x, y)*

*9: circle_range ← RADIUS ∗ clicknum ∗* (1/2)

*10:* **for** *(x_i_, y_i_) = (1, 1) ... (IMAGE_X_PIXEL, IMAGE_Y_PIXEL)* **do**

*11: length_from_center ← length((x_i_, y_i_), circle_center)*

*12:* **if** *length_from_center < circle_range* **then**

*13: intensity[x_i_][y_i_] ←*

*14: INTENSITY ∗ (circle_range – length_from_center)/circle_range*

*15:* **end if**

*16:* **end for**

*17:* **return** *intensity*

*18:* **end** **function**

*19:* **function** BodyMap_Graph*(p in ALL POINT )*

*20:* **for** *value = (low, mid, high)* **do**

*21: point_num_in_category ← 0*

*22:* **for all** *p(p.V ALUE = value)* **do**

*23: point_num_in_category ← point_num_in_category* + 1

*24: intensity_value ← intensity_value + Draw_Circle (p.X, p.Y, p.CLICK)*

*25:* **end for**

*26: intensity_value ← intensity_value/point_num_in_category*

*27: img[tag = value] ← DRAW (intensity_value)*

*28:* **end for**

*29:* **return** *img*

*30:* **end function**

**References**

1. Deng, Y., Loy, C. C. & Tang, X. Image aesthetic assessment: An experimental survey. IEEE Signal Process. Mag. 34, 80–106, DOI: 10.1109/msp.2017.2696576 (2017).
2. Li, L., Zhu, H., Zhao, S., Ding, G. & Lin, W. Personality-assisted multi-task learning for generic and personalized image aesthetics assessment. IEEE Transactions on Image Process. 29, 3898–3910, DOI: 10.1109/TIP.2020.2968285 (2020).
3. He, K., Zhang, X., Ren, S. & Sun, J. Deep residual learning for image recognition (2015). 1512.03385
4. Yang, Y. et al. Personalized image aesthetics assessment with rich attributes. In Proceedings of the IEEE/CVF Conference on Computer Vision and Pattern Recognition, 19861–19869 (2022)
